# Impact of Exposure to Leaves From Metal-Polluted Sites on the Developmental Parameters of Larvae of the Dark Sword-Grass, *Agrotis ipsilon* (Lepidoptera: Noctuidae)

**DOI:** 10.1007/s00244-024-01076-8

**Published:** 2024-07-24

**Authors:** Shrouk Rasmy, Amr Mohamed, Hesham A. Yousef

**Affiliations:** 1 Entomology Department, Faculty of Science, Cairo University, Giza, 12613 Egypt; 2 Research Fellow, Plant Protection Department, College of Food and Agricultural Sciences, King Saud University Museum of Arthropods, King Saud University, 11451 Riyadh, Saudi Arabia; 3 Division of Invertebrate Zoology, American Museum of Natural History, 200 Central Park West, New York, NY 10024 USA

## Abstract

Insects are impacted by pollutants in their environments and food sources. Herein, we set out a semi-field study to assess the impact of environmental heavy metal contamination on developmental parameters, energy reserves, and acidic and alkaline phosphatases in the larval *Agrotis ipsilon* (Lepidoptera: Noctuidae). Castor leaves from contaminated and uncontaminated (reference site) areas were fed to *A*. *ipsilon* larvae in all treatments. The heavy metal concentrations in the plant from different areas (contaminated and reference sites) and in the larvae were analyzed. Toxic effects were observed in the larvae feeding on the leaves from the metal contaminated areas. Larval and pupal weights, growth indices, and larval fitness were all significantly lower than in the reference group. Likewise, in the third and fourth instars, there was a significant decrease in both the survival and moth emergence rates. In contrast, the pupation duration was significantly longer. Total protein, lipid, and glycogen content showed significant reductions in treated larvae. Larval homogenate samples contaminated with heavy metals showed a significant increase in acid- and alkaline- phosphatase levels. The results obtained could provide a basis for a long-term evaluation of the risk associated with heavy metals and their impact on plant populations and important agricultural pests.

Heavy metal pollution severely affects various ecosystems, and the extent and severity of this pollution are rapidly increasing due to the mining, smelting, and burning of fossil fuels as well as the overuse of chemical fertilizers (Zhao et al. 2014; Angon et al. 2024). Heavy metal uptake by plants and successive accumulation in human tissues through the food chain cause human, animal, and environmental health concerns. One of the main concerns regarding heavy metals is soil pollution (Li et al. 2020; Zhang et al. 2020). Many metals, particularly Cu, Ni, Cd, Zn, Cr, and Pb, are responsible for heavy metal pollution of the soil (Karaca et al. 2010; Gall et al. 2015). Compared to organic pollutants, heavy metals have the characteristics of concealment, accumulation, and refractory degradation (Chalaris et al. 2023). They affect the quality of the atmosphere, soil, and water and transfer along the food chain to insects, humans, and other animals through their accumulation in plants, which can result in serious toxic effects (Galal and Shehata 2015). When concentrations of heavy metals exceed the regulatory ability of organisms, they will accumulate in tissues, potentially affecting behavior and life history (Jaishankar et al. 2014). A number of variables, such as feeding frequency, exposure duration, the body's capacity to absorb and eliminate heavy metals, and the mechanism underlying heavy metal toxicity, all have an impact on the degree of accumulation, which is a dynamic and ongoing process (Su et al. 2014).

The heavy metals available for plant uptake are those present as soluble components in the soil solution or those solubilized by root exudates (Blaylock and Huang 2000). Environmental heavy metals pollutants can cause oxidative stress to the insect (Yousef et al. 2019) by inducing redox reactions, the production of reactive oxygen species (ROS), and a series of other harmful consequences brought on by oxidative stress (Khalid et al. 2020). These species cause oxidation of proteins, damage to nucleic acids, lipid peroxidation and activation of the immune system (Hermes-Lima and Zenteno-Savıń 2002; Gavrilovic 2017). The increase of ROS impairs the absorption of nutrients and may cause oxidative damage in the midgut cells of insects.

Insects can accumulate heavy metals in their bodies when living in polluted areas (Zvereva et al. 2003; Soliman et al. 2022; Li et al. 2023). In those areas, insects can get into direct contact with contaminated soils in aquatic and terrestrial systems through air pollution and also through feeding on plants contaminated with heavy metals (Vickerman et al. 2002).

Brick kilns also make significant contributions to the heavy metal content of the soil (Haque et al. 2022). The acid deposition from the brick kiln creates a serious threat to human health as well, as it contaminates air, agricultural land, drinking water, and even food (Kumar et al. 2021). Helwan Company for Fertilizers and Chemical Industries (Cairo, Egypt) creates metal pollution, which accumulates in soil. It is common knowledge that an excess of metals in the environment can cause harm to the biota by altering the physiological activities (Yousef et al. 2010, 2017), including disruption of the reproductive potential and endocrine system (Drevnick and Sandheinrich 2003), immunosuppression (Carey and Bryant 1995), the induction of stress proteins (Piano et al. 2004) and oxidative stress (Farombi et al. 2007).

The dark sword-grass or black cutworm *Agrotis ipsilon* (Hufnagel, 1766) (Lepidoptera: Noctuidae), a well-known polyphagous pest of many vegetable crops in Egypt (EL-Safady et al. 2015), is a major insect pest that can destroy many important crops worldwide (Binning et al. 2015). Specifically, their larvae damage many crop species, vegetables, and weeds (CABI 2021). *Agrotis ipsilon* larvae can consume over 400 cm^2^ of foliage during their development (Capinera 2008), and were used as a model insect.

Our objective was to assess the detrimental effects of elevated environmental metals in food on specific developmental parameters in *A*. *ipsilon*. An additional objective of the study was to determine how metal pollutants (lead, chromium, zinc, nickel, and copper) affected the energy reserves (lipid, protein, and glycogen) and the acid and alkaline phosphatases in *A*. *ipsilon* larvae that were fed castor leaves that were collected from polluted areas. The analysis of the metal accumulation level in the *A*. *ipsilon* larval body may offer some insightful information about the bioaccumulation of heavy metals along the food chain (plant–insect).

## Materials and Methods

### Study Area

The study area is located east of the Nile River, 20 km south of Cairo, in the Al-Tebbin region of the Helwan Province, Cairo, Egypt. It is considered the biggest industrial zone in Egypt, with about 16.5% of the total industrial activities for the country. The main industrial activities include ferrous and nonferrous metallurgical work, a coke factory, chemicals, and the cement industry (Soliman et al. 2022, 2024).

### Insects

The *A. ipsilon* colony was originated from eggs obtained from the Pesticides Center, Faculty of Agriculture, Cairo University, Giza, Egypt. Castor leaves collected from both the polluted and non-polluted (as a reference) locations were fed to the larvae. To prevent cannibalism, the larvae in their 3rd instar were divided and transferred to plastic containers measuring height (h) 20 cm, depth (d) 20 cm, and width (w) 30 cm. The cutworm were reared at 27 ± 1°C, 10:14 L:D, and 60 ± 10% relative humidity (RH) after being transferred and divided into glass jars and given a 10% sugar solution (Archer et al. 1980).

### Sample Collection and Preparation

The castor bean, *Ricinus communis* L., leaves were collected from two locations in Egypt, referred to as sites A and B hereafter, with distinct pollution sources 600 m apart. Collection coordinates were reference site: 30.0264630, 31.2057960; site A: 29.7648721, 31.3114177; site B: 29.7482685, 31.3162675. The sources of pollution for sites A and B were determined to be brick kilns and Helwan Company for Fertilizers and Chemical Industries, Cairo, Egypt. For reference, the castor tree leaves were collected from Cairo University, Giza, Egypt, main campus gardens. Ultrapure water was used to clean the castor leaves in order to remove any impurities adhered to the surface. The leaves were collected, ground into a powder, and then dried in an oven at 70 °C to a consistent weight. They were then stored in falcon tubes. The sampling locations were recorded using Global Positioning System (GPS) technology. At a constant distance of 600 m from the emission source, samples were gathered. Group A is the larvae fed plant leaves from site A, group B is the larvae fed plant leaves from site B; and reference is the larvae fed plants brought from the reference site.

### Evaluation of *A. ipsilon* Individual Growth, Development, and Population Dynamics

About 60 eggs (for each replicate) were incubated at 27 °C and 60 ± 10% humidity. The larvae in their first instar were divided into groups, each group consisting of 40 individuals, and about 6 caps were used of sterile plastic containers measuring 20 cm in height and 30 cm in diameter. Each cap held 40 larvae, and they were fed fresh castor leaves every day that were collected from three locations: two polluted sites, A and B, and a reference site. Larvae were fed separately at the third stage of development to prevent cannibalism. The caps were regularly cleaned of excrement. For each group, the experiment was set up with five replicates, each containing sixty eggs. The adults that had emerged were given paper sheets to lay their eggs on and put in a jar with a screening cover so they could mate. A cotton swab was used to absorb a 10% sugar solution for adults. It was placed twice a day on the screening cover. Every morning after the oviposition began, paper sheets were removed. Data on moth emergence rate, pupation rate, pupal weight, death rate, and larval longevity were recorded for each life stage (Ali et al. 2019).

Using the formulas below from Pretorius (1976) and Itoyama et al. (1999), the growth and fitness metrics, such as the standardized growth index, pupal growth index, immature growth index, larval growth index, and fitness index, were calculated.

$$ {\text{Larval}}~{\text{growth}}~{\text{index}}~ = ~\frac{{Pupation\left( \%  \right)}}{{Larval~period~\left( {days} \right)}} $$

$$ {\text{Pupal}}~{\text{growth}}~{\text{index}}~ = ~\frac{{Emergence\left( \%  \right)}}{{Pupal~period\left( {~days~} \right)}} $$

$$ {\text{Immature}}~{\text{growth}}~{\text{index}}~ = \frac{{Emergence\left( \%  \right)}}{{\text{Im} maure~stages~\left( {days} \right)}} $$

$$ {\text{Standardized}}~{\text{growth}}~{\text{index~}} = ~\frac{{Pupal~weight}}{{Larval~period~\left( {days} \right)}} $$

$$ {\text{Fitness}}~{\text{index}} = \frac{{Pupation~\left( {~\% } \right)~x~Pupal~weight}}{{Larval~period~\left( {days} \right)~x~Pupal~period~\left( {days} \right)}} $$

$$ {\text{Larval}}~{\text{mortality}}~ = \frac{{number~of~death~from~first~to~sixth~instar}}{{number~of~total~larvae}} \times 100 $$

### Energy Reserves (Assays for Proteins, Lipids, and Glycogen)

A batch of 4 larvae on the 3rd day of 5th instar was used to obtain tissue homogenates. To obtain sample aliquots for assays, the larvae were homogenized at 24,000 rpm in phosphate buffer (50 mM, pH 7.4). Then, the homogenates were centrifuged at 10,000 x*g*. Phenylthiourea was added to suppress the development of melanin. The measurements were conducted using the obtained supernatant (Emre et al. 2013).

#### Tests for Protein Content

Protein contents were determined with the Bradford (1976) method, using Coomassie Brilliant blue G-250. Bovine Serum Albumin (BSA) was used as a standard. The absorbance of the supernatant was measured spectrophotometrically at 595 nm. The absorbance was measured by UV/Vis Jenway-7305 spectrophotometer (Bibby Scientific Limited, Staffordshire, UK).

#### Lipid Analysis

The total lipid content was determined with the Van Handel (1985) method with a slight modification. Four larvae were dissected, and gut contents removed to avoid interference with the energy stores in larval food homogenized in 8 mL of chloroform/methanol (1:2) and centrifuged for 10 min at 9,000 x*g*. A volume of 200 µL of the supernatant were pipetted into Eppendorf tubes; 960 µL of a vanillin phosphoric acid mixture and 40 µL of concentrated sulfuric acid were added to tubes, and the reddish color was allowed to develop for at least 5 min. The color is stable from 5 to 30 min, then slowly fades. The mixture was then measured spectrophotometrically at 525 nm using 0.1% soy oil as reference (Laura and Guiherme, 2005).

#### Glycogen Determination

Four larvae were homogenized in 10% TCA for five minutes for each aliquot preparation. The resulting pellet was then centrifuged at 24,000 rpm to be used in the determination of the glycogen amount. Glycogen was extracted with the Roe and Dailey (1966) method. Briefly, the pellet was mixed with ethyl alcohol and heated to 37 °C for 24 h. Then, the supernatant was collected by centrifugation at 3,500 x*g* for 30 min, and the alcohol was removed by incubating the solution at 37 °C. The Anthrone reagent (Roe and Dailey 1966) was used to measure the glycogen level, and color generation was measured spectrophotometrically at 620 nm (Emre et al. 2013). A standard of 0.1 mg/mL glycogen was employed.

### Tissue Preparation for the Assessment of Enzyme Activity

After dissecting the larvae, the midgut was extracted, and 2gm of midgut tissue were collected and homogenized on ice in 2 mL of buffer (0.1 M phosphate buffer, pH 7.2) that contained 20% glycerol, 1 mM phenylmethylsulfonyl fluoride (PMSF), 1 mM ethylenediaminetetraacetic acid (EDTA), and 1 mM dithiothreitol (DTT). After centrifuging the homogenate for 15 min at 4°C and 10,000 rpm, the supernatant was poured into a sterile Eppendorf tube, set on ice, and used right away to prepare acid phosphatase (ACP) and alkaline phosphatase (AKP). The method of Bradford (1976) was used to calculate the total protein content.

### Acid and Alkaline Phosphatase Assays

The method of Asakura (1978), with slight modifications, was employed in these assays. To assess the acid phosphatase activity, 50 µL of larval homogenate and 450 µL of pH 4.0, 50 mM sodium acetate buffer were mixed. 20 µL of larval homogenate were diluted to 500 µL with 50 mM Tris–HCl buffer at pH 8.0 and then mixed with an equivalent volume of the corresponding solution that contained 12.5 mM *p*-nitrophenyl phosphate to estimate the alkaline phosphatase activity. The enzymatic reaction was stopped by adding 100 µL of 0.5 N NaOH solution after the mixture was incubated for 15 min at 37°C in a water bath. The mixture was then centrifuged (4000 × g; 5 min). The absorbance of the clear supernatants was measured at 440 nm (Suganya et al. 2016).

### Determination of Metal Accumulation

The method outlined by Soliman et al. (2022) was used to estimate metal concentrations in leaves and larvae. The larvae and castor leaves were dried. All of the reagents and chemicals were analytical grade and were purchased from Merck. Before being used, all glassware and plastic materials were completely cleaned with ultrapure water, submerged in 2N nitric acid for an entire night, and then washed again with distilled water. A pre-digestion stage was conducted for 24 h at room temperature using 0.5 g of 4th instar larvae after 48h of molting (to ensure that the gut of the insect is empty of plant materials), 10.0 mL of 65% HNO_3_, and 2.0 mL of 30% H_2_O_2_ to break down the organic materials. The suspension was then heated on a thermostatically controlled hot plate at 90°C until it was almost dry in a fume hood. After adding 10 mL of double-distilled water to the heated residue, the walls of the flask were cleaned. The resulting suspension was then filtered through Whatman filter paper (No. 41) in a volumetric flask, diluted to 25 mL, and kept in polyethylene bottles at 4 °C for analysis. Using inductively coupled plasma atomic emission spectroscopy (ICP-AES; Horiba Jobin Yvon Ultima 2, France), the amounts of Cd, Cr, Ni, Cu, Pb, and Zn were measured. Calibration and accuracy verification standards were prepared using Agilent (Agilent Technologies, USA) and Spex (SPEX CertiPrep, USA) calibration standards. The wavelengths and detection limits of the ICP for the analyzed metals were: 226.502 nm and 0.0023 mg L^−1^ for Cd; 220.353 nm and 0.028 mg L^−1^ for Pb; 324.754 nm and 0.0036 mg L^−1^ for Cu; 213.856 nm and 0.0012 mg L^−1^ for Zn; and 205.552 nm and 0.0041 mg L^−1^ for Cr, respectively. For each analytical batch, an accuracy check of the measures was performed using a certified reference material (standard reference materials from the National Institute of Standards and Technology (NIST), USA: NIST 1547, 1577, and 2709 for plant, animal, and soil tissues, respectively) and a reagent process blank. The mean values of three replicates were calculated for each measurement. The concentration of metals was given in mg kg^−1^ dry weight.

### Bioconcentration and Translocation Factors

The calculation of the bioconcentration factor (BCF) was performed to assess whether insects could be categorized as accumulators, as follows:

$$ {\text{BCF~}} = \frac{{heavy~metal~in~in\sec t}}{{heavy~metals~in~castor~leaves}} $$

### Statistical Analysis

Heavy metal concentrations are presented as the mean ± SE. Data analysis was performed using a one-way analysis of variance (ANOVA, HSD test, *p *< 0.05). The data on life history were normally distributed according to the Kolmogorov–Smirnov test, *p *< 0.05. An ANOVA test was also applied to illustrate the effect of the site on the studied variables. *P *< 0.05 was considered a significant effect. The data were statistically analyzed using SPSS version 23 software.

## Results

### Heavy Metal Concentrations of Zn, Cu, Cr, Ni, Cd, and Pb Accumulated in the **Castor** Leaf Samples

**Fig. 1:**
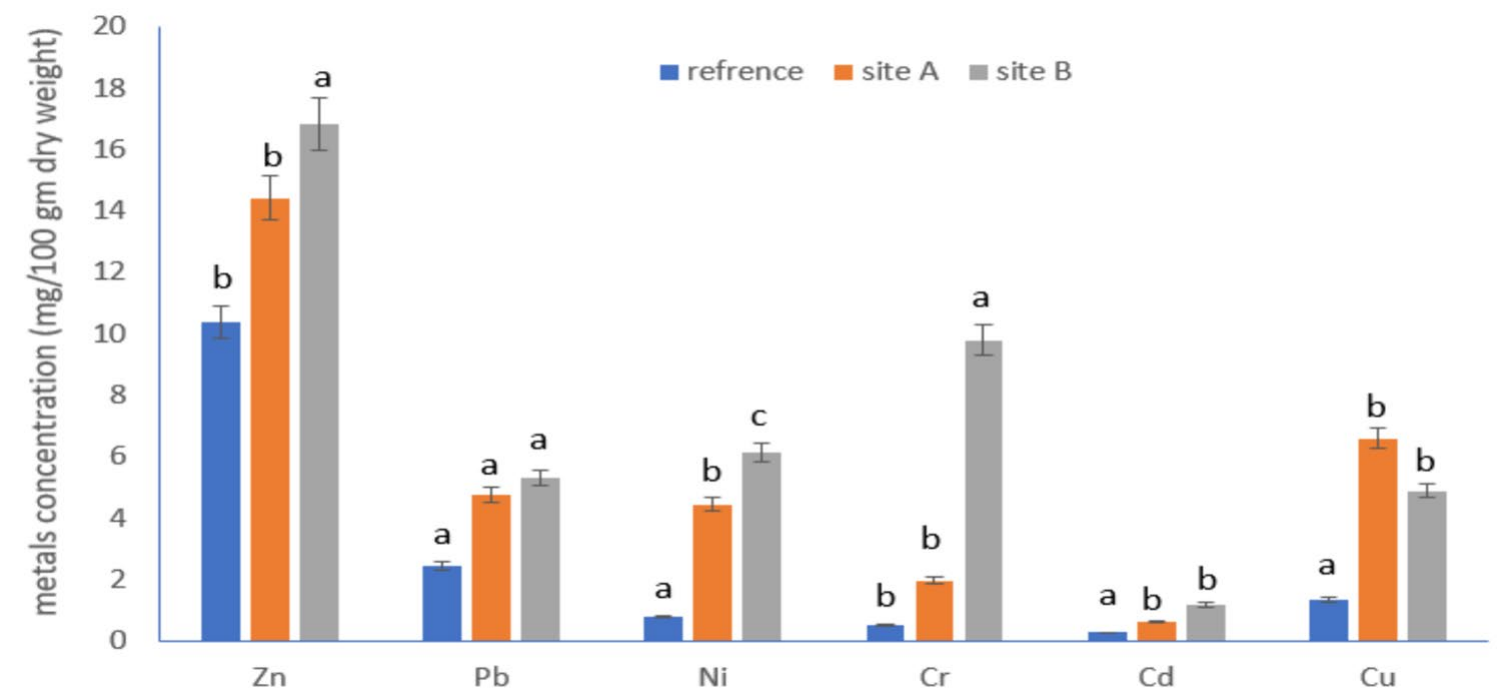
Concentrations of Zn, Pb, Ni, Cr, Cd, and Cu in the plant samples (castor leaves) collected at the polluted field sites (sites A and B) and reference site. Data are presented as mean ± SE. Among different sites, the means marked with the different letters are significantly different (*P*< 0.05), whereas those marked with similar ones are not significantly different (*P*> 0.05)

The concentration of heavy metals (mg/100 gm dry weight of plant leaves) in the plant samples obtained from the various locations varied noticeably (Fig. 1). There were no significant variations in Cu levels in the plant leaf samples between the two polluted sites A and B; however, the Cu concentration was considerably greater in the plant (castor leaves) sample from polluted sites (A and B) compared to the reference site (*P *= 0.025; F = 7.3). Out of all the heavy metals evaluated, the plant leaf samples had the lowest quantities of Cd among the other heavy metals examined, which is significantly higher in the leaves from polluted sites. Leaves from site B contained a significantly higher Cr concentration compared to those from reference site and site A (HSD test, ANOVA; *p *> 0.05) for reference site – site A data. In comparison to the reference site, Zn and Pb concentrations in the plant samples from site A were 1.6 and 2 times higher, respectively. The amount of Ni in the plant leaves differed significantly throughout the reference site and polluted sites (*P *= 0.006; F = 13.15) (HSD test, ANOVA; *p *< 0.05).

### Metal Body (mg/ 100 gm Dry Weight) Concentrations in the Insect Body of *A. ipsilon* Larvae Using ICP Analysis

**Fig. 2:**
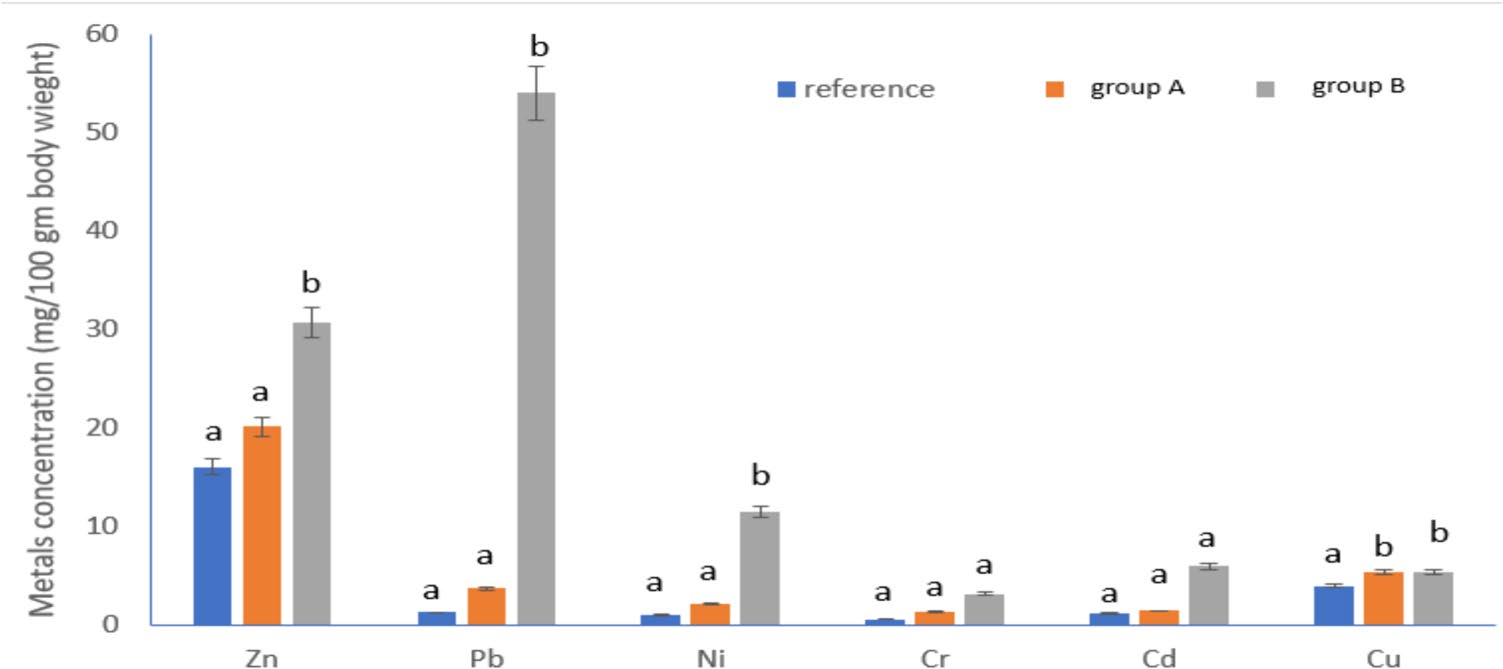
Concentrations of Zn, Pb, Ni, Cr, Cd, and Cu in the *A*. *ipsilon* larvae that fed plants from polluted sites and unpolluted sites (reference), where group A are larvae that fed plant leaves brought from site A, group B are larvae that fed plant leaves brought from site B and reference is the larvae that fed the pant leaves from the reference site. Data are presented as mean ± SE. Among different sites, the means marked with the different letters are significantly different (*P*< 0.05), whereas those marked with similar ones are not significantly different (*P*> 0.05)

The concentration of heavy metals (mg/100gm dry larval body weight) in the insect samples fed on leaves from the various locations varied noticeably (Fig. 2). Zinc metal is the highest concentration among the examined samples. In comparison to the larvae fed from reference site, the significant increase of metals (*P *< 0.05) were observed in the larvae fed from the site B (Group B). There were no significant differences observed in Cr and Cd metal concentrations in the larval body of different groups (Fig. 2).

### Effects on Larval Mortality and Pupation Rate of *Agrotisipsilon*

**Fig. 3:**
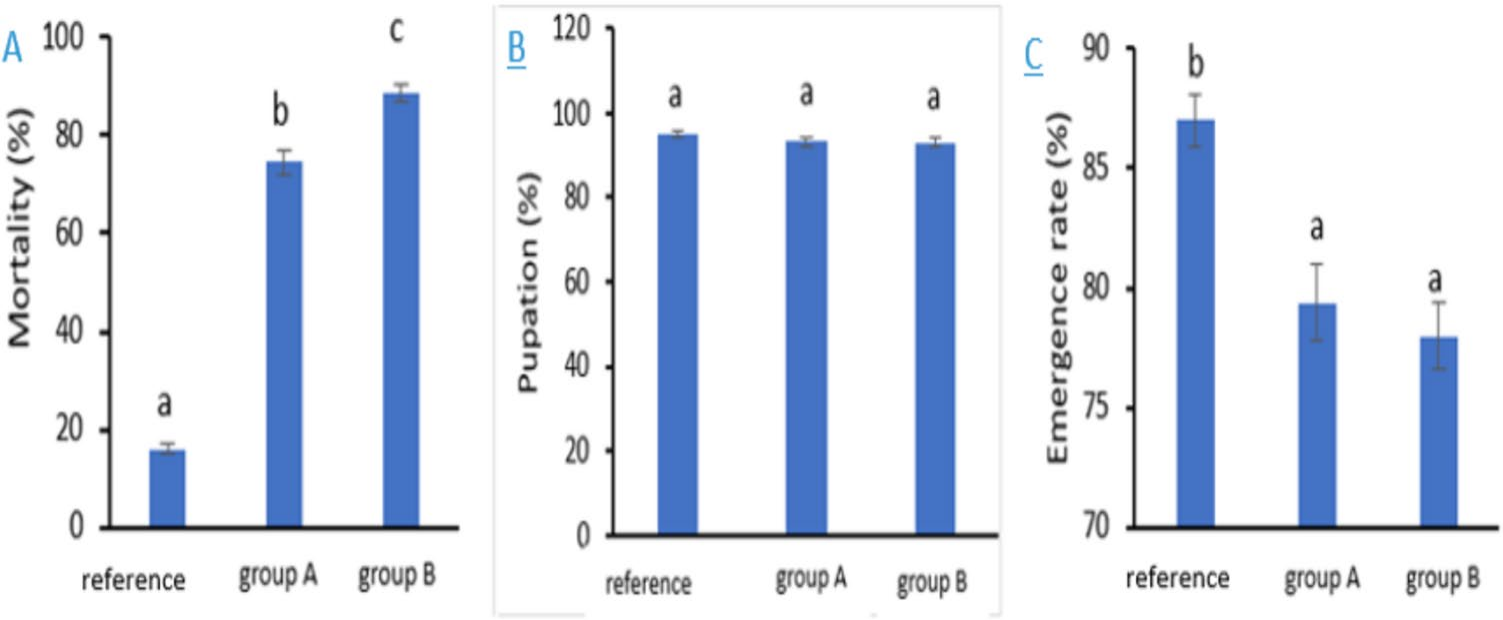
Mortality rate of larvae A, pupation rate B, and emergence rate C. Data are presented as mean ± SE. Among different groups, group A are larvae that fed plant leaves brought from site (**A**), group B are larvae that fed plant leaves brought from site (**B**) and reference is the larvae that fed the pant leaves from the reference site. The means marked with the different letters are significantly different (*P*< 0.05), whereas those marked with similar ones are not significantly different (*P*> 0.05)

Many of the *A*. *ipsilon* larvae fed leaves from the metal contaminated sites did not complete their life cycle, and there was greatly decreased larval survival and stunted larval growth, especially in the 3rd and 4th instars. The mortality rate of larvae fed on leaves from polluted sites was significantly higher than that fed on castor leaves from the reference site (F = 442.3, *P *< 0.001, Fig. 3A).

The larval mortality rate increased by 72.8% and 58.6%, respectively. The pupation rate of *A*. *ipsilon* is affected by contaminated food from sites A and B, although it is not substantially lower than the reference group (F2, 12 = 1.06, *P *= 0.377; Fig. 3B). The pupation rate in those groups dropped by 1.8% and 2%, respectively. The adult *A*. *ipsilon* moth emergence rate was likewise reduced by heavy metal pollution (F2, 12 = 12.215, *P *= 0.001; Fig. 3C). When compared to the reference site at these two groups, groups A and B both markedly lowered the *A*. *ipsilon* adult emergence rate by 7.6% and 9%, respectively.

### Effects on the Duration of Pupae, Pupal Mass, and Longevity of the Adult Moth

**Fig. 4:**
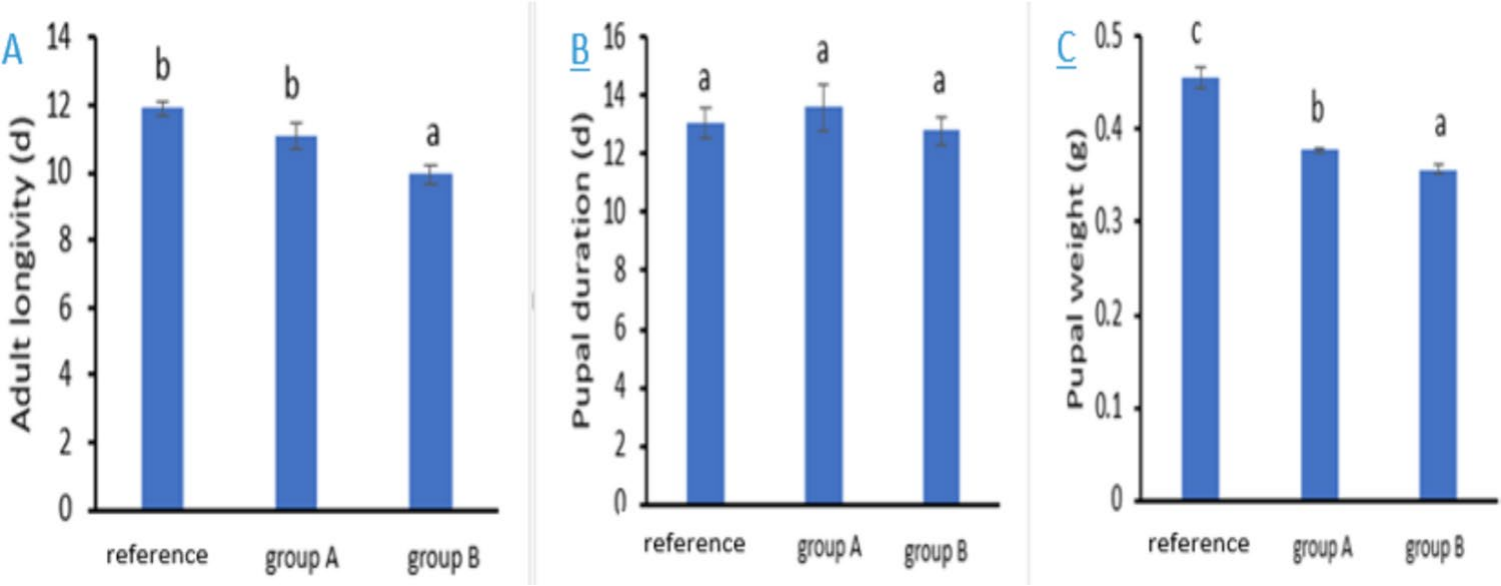
Adult longevity A, pupal duration B, and pupal weight C. Data are presented as mean ± standard error. Among different groups, group A are larvae that fed plant leaves brought from site (**A**), group B are larvae that fed plant leaves brought from site (**B**) and reference is the larvae that fed the pant leaves from the reference site. The means marked with the different letters are significantly different (*P *< 0.05), whereas those marked with similar ones are not significantly different (*P *> 0.05)

The *A*. *ipsilon* adult stage (i.e., moth) was significantly impacted in the treatments where larvae were fed leaves from the metal contaminated sites (F2, 27 = 9.97, *P *= 0.001, Fig. 4A). When fed on leaves taken from sites A and B, the longevity of the larvae was significantly shorter than at the reference site (by 0.8 and 1.95 days, respectively). In both groups A and B, the pupal duration was longer, however, the difference was not statistically significant when compared to the reference site (F2, 12 = 442.3, *P *= 0.676) (Fig. 4B).

When compared to larvae fed on leaves from the reference site, the pupal length duration of those fed on castor leaves from contaminated locations (A and B) increased significantly by 0.5 and 0.3 days, respectively. Furthermore, we found that the pupal weight of the *A*. *ipsilon* was significantly impacted by heavy metals (F2, 57 = 61.39, *P *< 0.001, Fig. 4C).

### The Impact on the Duration of *Agrotisipsilon* Larval Stages

#### Duration of Larval Phase

When compared to the treatment from the reference site, the treatments with leaves from the metal contaminated site had a substantial impact on the length of time the larvae spent (in days) in each stage (Table 1). When compared to the reference group, contaminated plant leaves from site B dramatically shortened the larval duration period.

**Table 1:** Larval instar duration of larvae fed on plants collected from three distinct sites (two contaminated areas, A and B, and the reference site)

Instar	Instar period fed on plant collected from			ANOVA
	Reference group	group A	group B	
1st	5.80 ± 0.37^a^	5.40 ± 0.24^a^	5.60 ± 0.24^a^	F_2,12_ = 0.655, *P *= 0.537
2nd	5.40 ± 0.24^b^	3.60 ± 0.24^a^	4.60 ± 0.40^b^	F_2,12_ = 8.714, *P *= 0.005
3rd	3.60 ± 0.24^a^	3.20 ± 0.20^a^	3.60 ± 0.24^a^	F_2,12_ = 1.81, *P *= 0.206
4th	4.20 ± 0.20^b^	3.40 ± 0.24^a^	3.60 ± 0.24^ab^	F_2,12_ = 3.25, *P *= 0.074
5th	5.60 ± 0.24^b^	3.80 ± 0.58^a^	3.60 ± 0.24^a^	F_2,12_ = 6.741, *P *= 0.011
6th	7.80 ± 0.20^b^	6.20 ± 0.20^a^	6.60 ± 0.24^a^	F_2,12_ = 20.818, *P *= 0.0001
Prepupal	1.40 ± 0.24^a^	1.00 ± 0.001^a^	1.20 ± 0.20^a^	F_2,12_ = 0.961, *P *= 0.41
Total	33.80 ± 0.37^b^	29.20 ± 0.20^a^	29.40 ± 0.24^a^	F_2,12_ = 84.50, *P *= 0.000

The data is shown as days ± SE. The means of the various groups—group A being the larvae fed plant leaves brought from site A, group B being the larvae fed plant leaves brought from site B, and reference being the larvae fed pant leaves from the reference site—are significantly different (*P *< 0.05) from one another, while those marked with similar superscript letters are not significantly different (*P *> 0.05)

The larval duration of the 2nd instar was significantly impacted by feeding with leaves from the metal contaminated site (F2, 12 = 8.714, *P *= 0.005, Table 1). Compared to reference group, the larval duration of *A*. *ipsilon* fed on castor leaves from polluted areas (A and B) was significantly shorter by 1.8 and 2 days, respectively for the 5th instar larvae. Furthermore, the larval duration in the 6th instar significantly decreased when feeding on plants from polluted sites A and B in comparison to data from non-polluted or reference site (F2, 12 = 20.818, *P* = 0.0001). However, the data in Table 1 reveal that the treatment with contaminated leaves did not induce a significant effect on the length of the pre-pupation stage of the larvae (F2, 12 = 0.961, *P *= 0.41). Moreover, it took 4.6 days and 4.4 days for larvae to develop into pupae when fed on castor leaves from sites A and B, respectively, except for the prepupal period (no effect).

Bioconcentration factors of greater than 1 can be found for Zn in all groups (F = 326.123, *p *< 0.001). While Pb the bioconcentration factor, was greater than 1 in group B only (*P*< 0.001). Metal content varied significantly among sample sites for Cu (F = 375.9, *p *< 0.001) and Cd (F = 578.05, *p*< 0.001) (Table 2).

**Table 2:** Bioconcentration factors of heavy metals in *A. ipsilon* larvae

Feeding insect groups	Zn	Pb	Ni	Cr	Cd	Cu
Reference group	1.55 ± 0.09^a^	0.54 ± 0.04^b^	1.35 ± 0.04^a^	1.15 ± 0.04^a^	4.06 ± 0.09^a^	3.05 ± 0.12^a^
Group A	3.45 ± 0.14^b^	0.78 ± 0.12^b^	0.49 ± 0.01^b^	0.72 ± 0.05^b^	2.36 ± 0.05^b^	0.82 ± 0.008^b^
Group B	1.83 ± 0.04^c^	9.4 ± 0.21^a^	1.89 ± 0.08^c^	0.33 ± 0.02^c^	5.14 ± 0.14^c^	1.11 ± 0.15^c^

Data are presented as mean ± SE of metal concentration in the insect body/mean of metal concentration in castor leaves. Group A consisted of larvae that fed plant leaves brought from site A, Group B consisted of larvae that fed plant leaves brought from site B, and the reference group consisted of larvae that fed the pant leaves from the reference site. This information was based on feeding plant leaves from different sites. The means denoted by different superscript letters differ significantly (*P *< 0.05), whereas the means denoted by the same ones do not differ significantly (*P *> 0.05)

### Effects on the Body Weight of *A. ipsilon* Larvae at Each Stage of Development (instars)

**Fig. 5:**
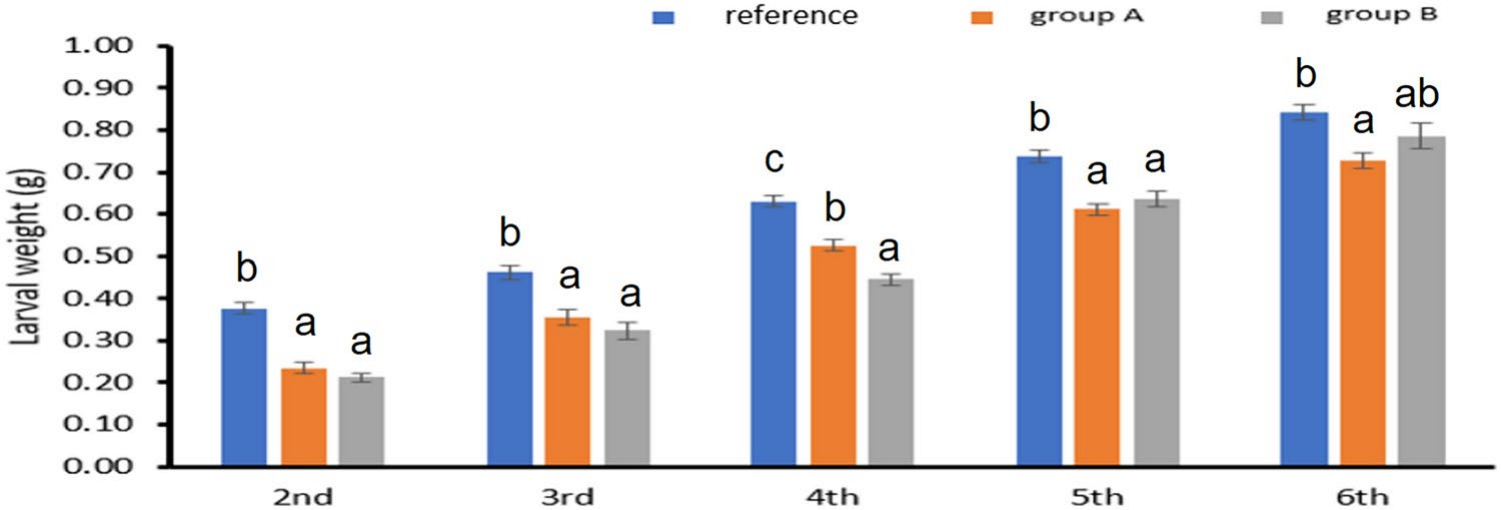
Larval weight (g). Data are presented as mean ± standard error. Based on feeding plant leaves from different sites, group A are larvae that fed plant leaves brought from site A, group B are larvae that fed plant leaves brought from site B and reference is the larvae that fed the pant leaves from the reference site. The means marked with the different letters are significantly different (*P *< 0.05), whereas those marked with similar ones are insignificantly different (*P *> 0.05)

The larval body weight was significantly impacted in the treatments with metal contaminated leaves (Fig. 5). When compared to the reference site, the larvae fed on leaves from sites A and B had a considerable decline in body weight throughout their second instar.

In comparison to the reference group (F2, 27 = 19.05, *P*< 0.001), the fifth instar showed a significant decline in body weight (F2, 27 = 50.72, *P*< 0.001). Additionally, compared to the reference group, the 6th instar fed on leaves from polluted areas had a significantly lower body mass (F2, 27 = 6.23, *P*= 0.006); at groups A and B, the reductions in body mass were 0.11 and 0.05 gm, respectively.

### Effects on *A.ipsilon* Larvae Mortality at Each Instar

**Fig. 6:**
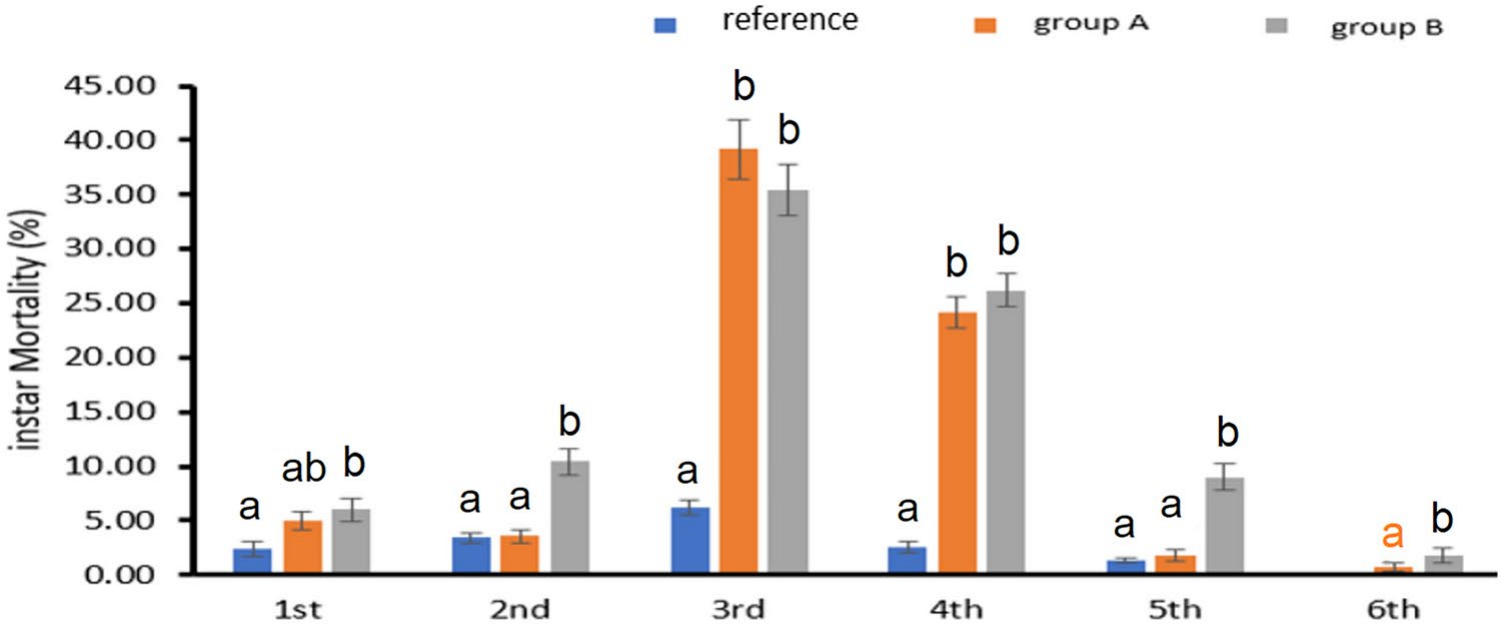
Instar mortality rate. Data are presented as mean ± standard error, where group A are larvae that fed plant leaves brought from site A, group B are larvae that fed plant leaves brought from site B and reference is the larvae that fed the pant leaves from the reference site. Among different sites, the means marked with the different letters are significantly different (*P *< 0.05), whereas those marked with similar ones are not significantly different (*P *> 0.05)

The larvae of *A*. *ipsilon* were not entirely prevented from completing their life cycle by heavy metal contamination. However, there was a significant drop in the 3rd and 4th instars' survival rate (Fig. 6).

In comparison to larvae fed from the reference site, the 3rd instar mortality rate of larvae given castor leaves from polluted sites A and B was considerably higher (F = 72.77; *P*< 0.001). When compared to the reference site, the mortality rate of the fourth instar was considerably higher at groups A and B (F = 110.72; *P*< 0.001). Furthermore, compared to reference site, the third instar mortality rate increased dramatically for groups A and B, rising by 33% and 29.2%, respectively. The mortality rate of older instars (5th and 6th) was at its minimum in the 6th instar; all larvae passed to the prepupal stage with no mortality (F = 28.88, *p*< 0.001) and (F = 4.207, *p*= 0.04), respectively.

### Growth and Fitness Indices

**Fig. 7:**
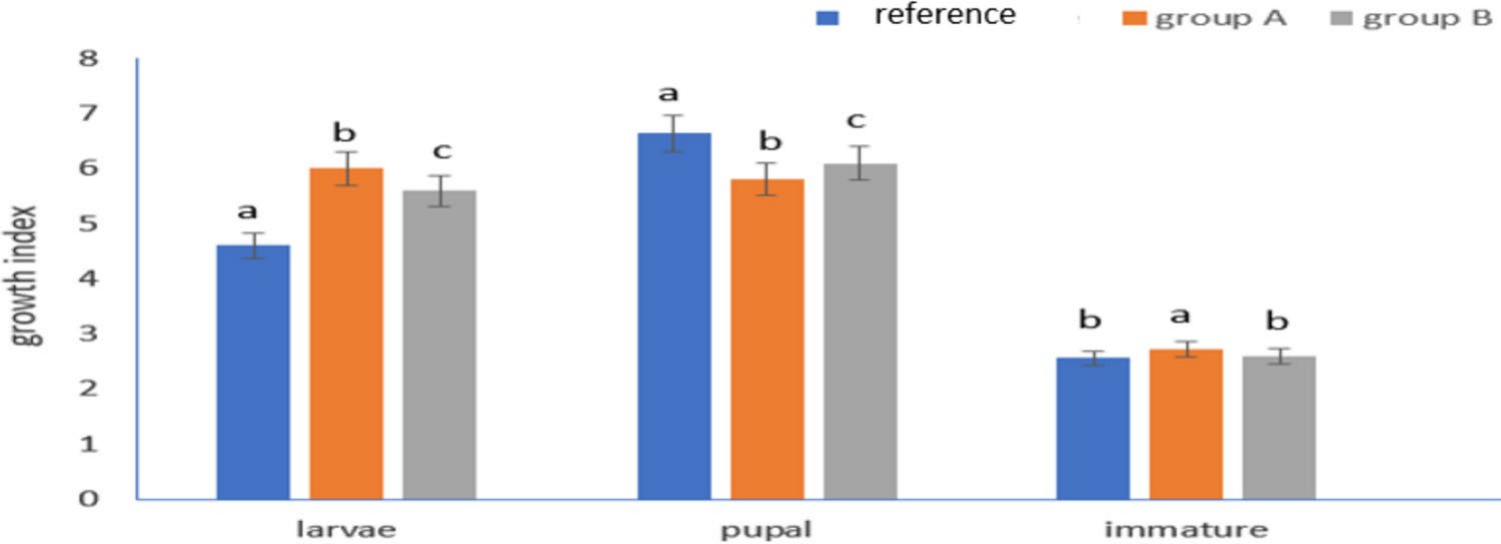
Larval growth index, pupal growth index, and immature growth index. Where group A are larvae that fed plant leaves brought from site A, group B are larvae that fed plant leaves brought from site B and reference is the larvae that fed the pant leaves from the reference site. Data are presented as mean ± SE. Among different sites, the means marked with the different letters are significantly different (*P *< 0.05), whereas those marked with similar ones are not significantly different (*P *> 0.05)

Treatments with leaves from the metal contaminated sites significantly affected (*P*< 0.001) larval growth, pupal growth, and immature growth of *A. ipsilon* larvae (Fig. 7). The larval growth index was at its maximum (6) in larvae fed on leaves from site A, while the lowest value (4.6) was observed when larvae were fed castor leaves from the reference site. The pupal growth index of *A. ipsilon* was the highest (6.64) at the reference site, while the lowest value (5.8) was observed when larvae were fed on plant leaves from site A (Fig. 7).

In the cases of group A larvae, the immature growth index (2.83) was significantly different compared to reference site (2.68), but in group B, the immature growth index (2.65) was not significantly different from the reference site treatment (Fig. 7). Similar results were also found in the case of the standardized growth and fitness index. The reference group showed the highest values of standardized growth (0.022) and fitness. Which may be attributed to the larval duration period, whereas growth index and immature index are strongly related to the immature lifetime (days).

### **Impact** on Total Contents of Energy Reserves (Protein, Lipid and Glycogen) in *Agrotis ipsilon* Larvae

**Fig. 8:**
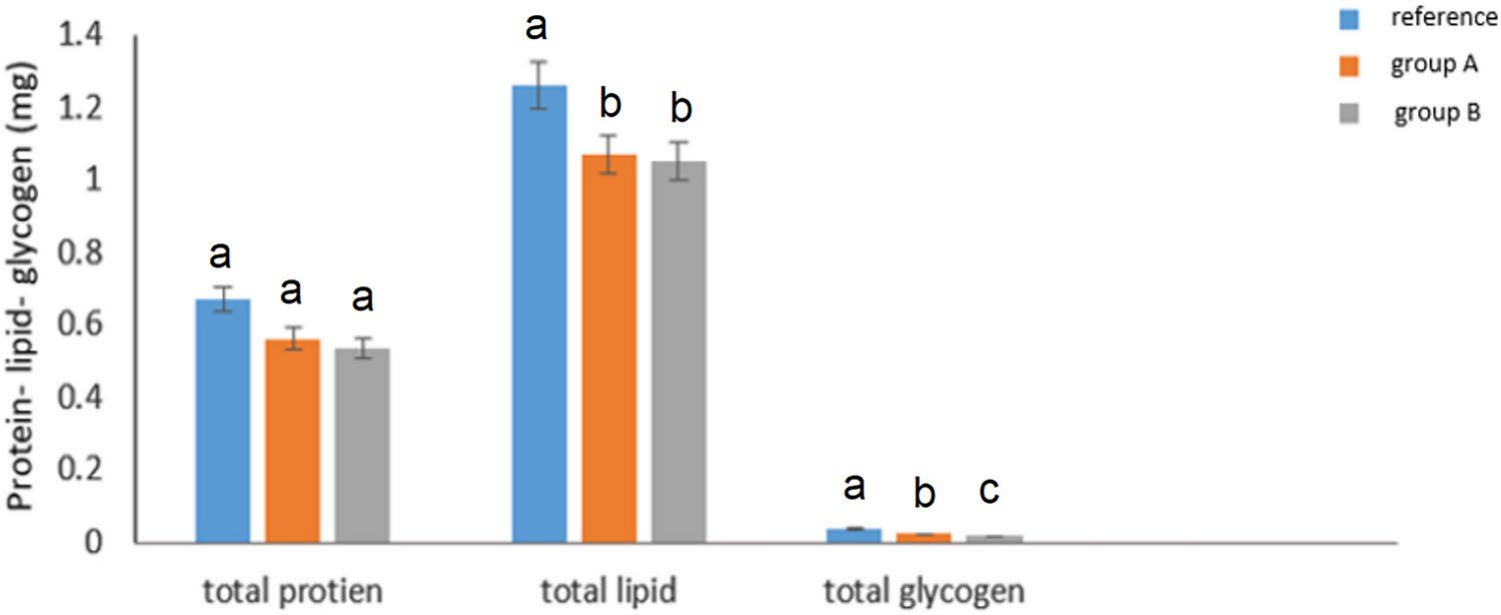
Total protein, lipid, and glycogen amounts. where group A are larvae that fed plant leaves brought from site A, group B are larvae that fed plant leaves brought from site B and reference is the larvae that fed the pant leaves from the reference site. Data are presented as mean ± SE in mg/ 2 g total fresh body weight. Based on feeding plant leaves from different sites, the means marked with the different letters are significantly different (*P *< 0.05), whereas those marked with similar ones are not significantly different (*P *> 0.05)

The effects of treatments with metal contaminated leaves on the total protein, lipid, and glycogen content of larval *A*. *ipsilon* are shown in Fig. 8 (F = 2.66; *p*= 0.149). After feeding on contaminated plants from sites A and B, the protein content dropped to 0.563 mg and 0.535 mg, respectively. Initially, the protein content was 0.673 mg. When compared to the reference group, the lipid content of individual larvae fed from sites A and B considerably decreased (F = 27.2; df = 2; *p*= 0.001). When compared to the reference group, the larvae fed from sites A and B (groups A & B) had a substantial decrease in glycogen (F = 237.0; df = 2; *p*< 001).

### Effects on AKP Assays of *A.ipsilon* Larvae

**Fig. 9:**
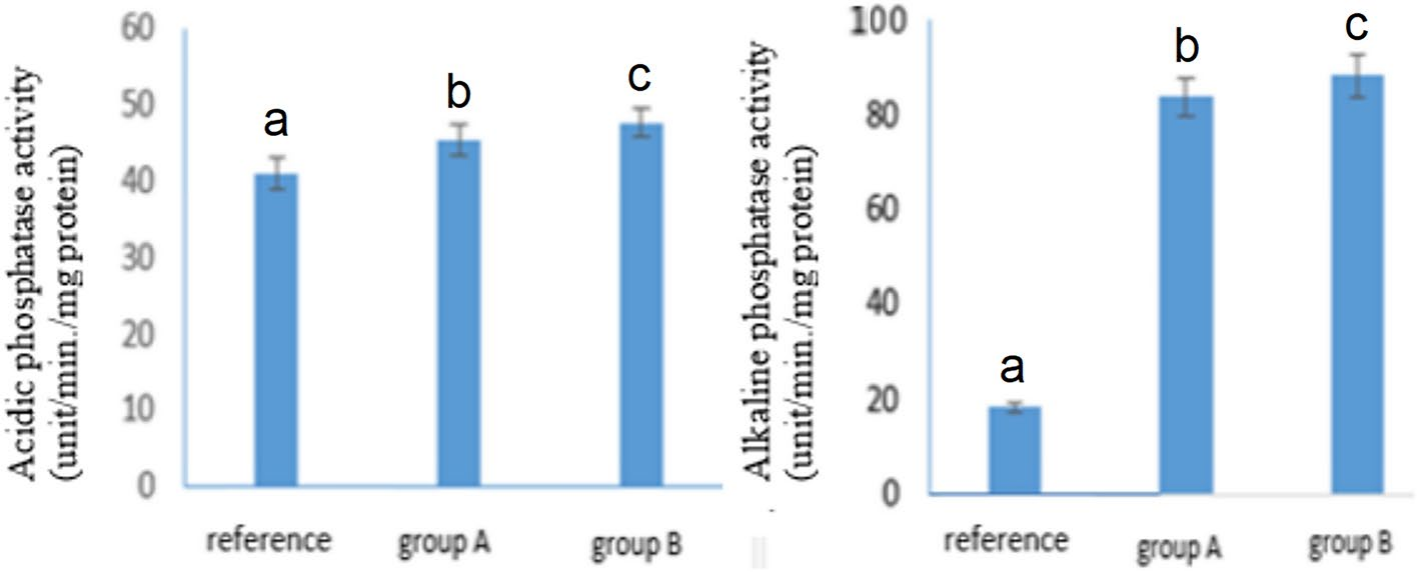
Acidic phosphate level and alkaline phosphate levels in the midgut tissues of larvae fed on castor leaves obtained from reference sites and polluted sites (A and B), where group A are larvae that fed plant leaves brought from site A, group B are larvae that fed plant leaves brought from site B and reference is the larvae fed the pant leaves from the reference site. Data are presented as mean ± standard error. The means marked with the different letters are significantly different (*P *< 0.05), whereas those marked with similar ones are not significantly different (*P *> 0.05)

The midgut tissues of *A*. *ipsilon* fed on castor leaves from polluted sites (A and B) exhibit considerably higher acidic phosphatase activity when compared to the reference group (F = 144.77; df = 2; *p*< 0.001). The midgut tissue of larvae fed from sites A and B exhibited a substantial increase in alkaline phosphatase activity as compared to the larvae fed from the reference site (F = 65,702.29; df = 2; *p*< 0.001), as illustrated in Fig. 9.

## Discussion

Heavy metal environmental contamination is a global health concern (Tchounwou et al. 2012; Chang and Cockerham 2019). Certain heavy metals, like Fe, Cu, and Zn, are essential trace nutrients for many biological processes, including the regulation of nerve transmission, the synthesis of cellular energy, and the transfer of oxygen, and many more. Furthermore, Cu catalyzes the defense of the cell against oxygen radicals as well as the reactions of oxidation and reduction (Krężel and Maret 2017). Translation, transcription, and DNA replication all depend on Zn. It regulates how hundreds of different enzymes function correctly and how proteins are put together. In addition to their roles in signal transduction, metals have a crucial regulatory role in the neurological system (Takeda 2000; Maret 2005). Both zinc and copper are necessary metals that also link to the cytosol metallothionein in the midgut of many species, but in excess, they can be poisonous (Lu et al. 2011), whereas metallothioneins are tiny, non-specific metal binding proteins that bind both necessary and unnecessary metal ions and transfer them outside the cell for detoxification (Slobodianet al. 2021). Some heavy metals are not necessary for living organisms, like Cd, which can enter cells even at low concentrations. They have the ability to impact an organism's ability to grow, develop, and reproduce (Schmidt et al. 1992; Mireji et al. 2010). After being absorbed, heavy metals can build up and become more concentrated in the food chain, harming the organismal metabolism and genetic components (Huang et al. 2012). Insect growth (Warrington 1987), mortality (Mitterbock and Fuhrer 1988), and physiology (Ilijin et al. 2009) are all clearly impacted by heavy metals. Cadmium can cause mutations in an organism and is highly toxic, even at low concentrations (Emre et al. 2013). Heavy metals can have a variety of effects on the relative growth rate (RGR). For example, lipids and proteins are important energy sources and affect insect populations. In *Spodoptera litura*, low concentrations of Ni increased RGR, while high concentrations of Ni decreased RGR (Sun et al. 2011). In insects, energy is stored as proteins, lipids, and carbohydrates for synthesis or breakdown into energy via a variety of pathways known as intermediary metabolism (Arrese and Soulages 2010).

The concentration and stress duration of heavy metals in food and the concentration of heavy metals in insects are closely correlated (Tőzsér et al. 2019; Soliman et al. 2024). For instance, the accumulation of heavy metals in the bodies and tissues of *Prodenia litura* after feeding them meals containing Cd and Pb corresponded favorably with the concentration in the food (Shu et al. 2015; Ding et al. 2013). The age of the midge larvae *Chironomus kiiensis* and *C*. *javanus*, the kind of metal (Pb or Cd), and the length of time the larvae were exposed to the metals all had an impact on the toxic effects of the metals on larvae (Ebau et al. 2012). Studies on *Oxya chinensis* under Cd stress (Zhang et al. 2011) and *Bembidion lampros* under Zn stress (Winder et al. 1999) yielded similar results. Furthermore, *D. melanogaster* Meigen larval survival rate declined when exposed to the heavy metal Cd (Shirley and Sibly 1999). The weight, rate of pupation, and development of the larvae of *Ceratitis capitata* were adversely impacted by the rise in metal (Cu, Cd, and Pb) concentrations (Kazimirova et al. 1997).

The rates of larval survival, pupation, and adult emergence of *S*. *litura* across multiple generations have been found to decrease in response to an increase in heavy metal Ni stress in previous studies (Sun et al. 2007). Additionally, the survival rate of D. *melanogaster* larvae exposed to the heavy metal Cd decreased (Shirley and Sibly 1999). The pupal weight, pupation rate, and larval development of *Ceratitis capitata* were negatively impacted by increasing concentrations of metal (Cu, Cd, and Pb) (Kazimirova et al. 1997).

The present work revealed that feeding *A*. *ipsilon* larvae on plant leaves with high concentrations of heavy metals may stimulate the growth of early larvae even though metals accumulate in the body during the 4th and 5th instars. Due to the high concentration of heavy metals, there was a lag in development that primarily affected the later stages (pupation and moth emergence). This resulted in shorter larval duration, increased larval mortality, and decreased growth and fitness indices. It cannot be ruled out that other factors, such as differences in the nutritional content of the castor leaves collected at the contaminated and reference sites may have contributed to the observed effects. The value of this present study is that effects were shown in treatments from contaminated leaves collected in the field, rather than contaminated leaves cultivated in the laboratory. However, this study design cannot control all the factors that may confound the study results, such as differences in the nutritional content of the leaves.

Higher amounts of heavy metals may denaturize proteins, which can lead to increased dysfunction in *A*. *ipsilon*. Certain copper concentrations (50 mg/kg) may make some animals hungrier and increase their nutritional intake (Kim et al. 2022). Copper may also enhance the microstructure of the intestines, inhibit the growth of pathogenic bacteria, improve enzyme function, and strengthen the immune system (Huang et al. 2012). According to Kim et al. (2022) observations, we infer that Cu included in food could help early *A*. *ipsilon* larvae develop and survive. However, it is plausible that the *A. ipsilon* larvae continuous ingestion of contaminated food containing heavy metals resulted in the build-up of metals, which is what caused the lower survival rates in the later instars, during pupation, and during moth emergence. The findings of earlier studies (Larsen et al. 1994; Kazimirova et al. 1997; Shirley and Sibly 1999; Sun et al. 2007) were comparable to this assertion. The Helwan Company for Fertilizers and Chemical Industries and brick kilns were the two distinct sites with two separate pollution sources from which the biochemical impacts of environmental contaminants on the insect *A*. *ipsilon* were researched in the current paper. Even yet, fertilizer industrial operations have a noticeable and substantial impact on the area around them. In comparison to the reference site, the samples from sites A and B consistently had greater quantities of heavy metals (Cd, Ni, Cr, Pb, Cu, and Zn) (Fig. 1). Meanwhile, it was not evident how the locations of the sites and the levels of pollutants in the plant samples related to each other. Due to the small differences in the plantings between the sites, there might have been some variations. Clay, minerals, and organic materials are examples of components that can alter the dynamics of pollution uptake (Angon et al. 2024). Oxidative stress and increased generation of reactive oxygen species (ROS) in exposed cells can be brought on by fertilizer industrial waste products such as metals, phosphates, sulphates, and dust (Okamoto et al. 2014; Zhu et al. 2014).

Norton and Kench (1977) have demonstrated how some heavy metals, such as Cd, prevent the production of proteins in vitro. *Pimpla turionellae* (L.) (Ortel 1991), *Sphaerodema urinator* (Dufour) (Bream 2003), and *Boettcherisca peregrina* (Robineau-Desvoidy) (Wu et al. 2006) all showed similar findings in in vivo investigations. Our study has demonstrated that *A*. *ipsilon* protein content is considerably reduced when exposed to heavy metal contamination. This could be the consequence of either a reduction in lipoproteins, which are used to repair damages produced by hazardous chemicals (Sancho et al. 1998) or direct inhibition of protein synthesis (Norton and Kench 1977). To manage the stress brought on by heavy metals, energy metabolism is essential (Wu et al. 2006). Glycogen storage is indicative of the level of contamination (Lagadic et al. 1994) in tissues with high metabolic and physiological activity (Wilps and Gade 1990). In our investigation, the amount of glycogen decreased dramatically when heavy metal contamination was present. Comparable findings were noted for *Nucella lapillus* (L.) (Leung and Furness 2001) and *Lymantria dispar* (L.) larvae (Bischof 1995). Bischof (1995) reported that trehalose and glycogen in hemolymph and total body tissue were significantly reduced in parasite larvae exposed to heavy metals. Both Spring et al. (1977) and Moura et al. (2000) linked this with the suppression of oxidative phosphorylation and increased glycolysis as an adaptive response to heavy metal stress. The decrease of lipid levels in our study (Fig. 8), which is linked with concentrations of heavy metals, is corroborated by the results of *G*. *mellonella* (Byung-Sik et al. 2001), *P*. *turionellae* (Ortel 1991), *L*. *dispar* (Bischof 1995), and *B*. *peregrina* (Wu et al. 2006). This shows that in stressful circumstances caused by heavy metal contamination, lipids can support other energy sources to meet the elevated energy requirements. These reductions in protein, glycogen, and lipid levels could be the consequence of the heavy metal-polluted insect not utilizing its nutrients to the fullest extent possible because heavy metals affect the absorption and digestion of nutrients (Fountain and Hopkin 2001).

Heavy metals are commonly found in roots and leaves. The process of heavy metal binding in plants occurs via the mechanisms of heavy metal accumulation and tolerance (Skuza et al 2022). As in mangrove roots, they can thus contribute to the prevention of heavy metal accumulation in the surrounding environment; high concentrations of heavy metal contaminants in sediments may be stored/ extracted by the tissues and leaves of trees (Hossain et al. 2022). In the Biesbosch floodplains, Notten et al. (2005) suggested that metal transfer from plant leaves to the snail is more important than metal transfer from the soil. Likewise, in our study, strong relationships were found between the plants and *A*. *ipsilon* larvae for all examined metals, and this may confirm the expectation that food is the primary factor determining metal bioaccumulation in animals (Notten et al. 2005; Chary et al. 2008; Zhang et al. 2011). Our study showed that Cd, Cu, Pb, and Zn accumulated in the cutworm larvae, with Pb having the highest bioaccumulation factor. The influence of homeostatic regulation on metal concentrations in organisms may explain the larvae's low accumulation of some heavy metals. In general, uptake, accumulation, and excretion rates of these essential metals in terrestrial animals are regulated by metallothionein and metallothionein-like proteins (Nfon et al. 2009; Newman 2014), and, based on the physiological requirements of these elements in the organisms, their bioaccumulation in the food chain is detected. Usually, naturally occurring concentrations of Ni seem to be sufficient to meet intake requirements because of their low physiological requirements (Phipps et al. 2002). However, essential metals, such as Zn, are usually biomagnified up the food chain through invertebrates that are known to be efficient accumulators or that lack the necessary regulatory and detoxification mechanisms of higher-order animals (Nfon et al. 2009).

The present work offers some indications of the efficiency of the *A. ipsilon* larvae in accumulating different trace elements. It also provides additional evidence for metal bioaccumulation in the terrestrial food chain. As major consumers, *A. ipsilon* larvae accumulated heavy metals in their bodies. The related study's findings showed that metal Cd, Cu, Pb, and Zn concentrations in the larvae's bodies were higher than in the plants (e.g., Devkota and Schmidt 2000; Zheng et al. 2008; Zhang et al. 2014).

ACP and AKP participate in the transport and digestion of nutrients as well as helping, modifying, and speeding up phagocytosis (Tian et al. 2023). Besides the role of AKP in dephosphorylating the nutrients, it also plays a part in the modulation of insect developmental hormones, juvenile hormone, and 20-hydroxyecdysone (Rauschenbach et al. 2007) and an unexpected role in the regulation of insecticide resistance (Wang et al. 2011). When comparing the ACP activity of metal-treated *A*. *ipsilon* midgut tissues to reference one, there was a significant increase (*P*< 0.001) observed in larvae fed contaminated plants from polluted sites A and B. The midgut tissues of *A*. *ipsilon* treated with metal exhibit considerably higher activity in the AKP as compared to the reference one. However, when the concentration of heavy metals increased, the exposure to heavy metals such as Cr, Pb, ACP, and AKP activities was dramatically reduced (Rodrick 1979).

## Conclusion

The study addresses dietary metal toxicity in *Agrotis ipsilon*, an agricultural pest. The study provides an understanding of the prospective flow of heavy metals from the plant to the insect pest, assessing their impact and toxicity, and enabling a conclusion about the tolerance to heavy metals. The lethal impact of heavy metal can also be inferred. Increased ACP and APP activity, along with metabolic fluctuations in carbohydrates, proteins, and lipid content, may indicate that the insect pest has adapted to metal stress. The use of field-sourced, contaminated diets adds environmental realism to the study. Previous studies and findings on dietary toxicity are generally confirmed. To decrease the impact of human activity on metal toxicity, immediate action is therefore required. Implications for pesticide management strategies may arise from the study's findings.

## References

[CR1] Ali S, Ullah MI, Saeed MF, Khalid S, Saqib M, Arshad M, Afzal M, Damalas CA (2019) Heavy metal exposure through artificial diet reduces growth and survival of *Spodoptera litura* (Lepidoptera: Noctuidae). Environ Sci Pollut Res 26(14):14426–14434. 10.1007/s11356-019-04792-0 10.1007/s11356-019-04792-0 30868456

[CR3] Angon PB, Islam MS, Kc S, Das A, Anjum N, Poudel A, Suchi SA (2024) Sources, effects and present perspectives of heavy metals contamination: soil, plants and human food chain. Heliyon 10(7):e28357. 10.1016/j.heliyon.2024.e28357 38590838 10.1016/j.heliyon.2024.e28357 PMC10999863

[CR4] Archer TL, Musick GL, Murray RL (1980) Influence of temperature and moisture on black cutworm (Lepidoptera: Noctuidae) development and reproduction. Can Entomol 112:665–673. 10.4039/Ent112665-7 10.4039/Ent112665-7

[CR5] Arrese EL, Soulages JL (2010) Insect fat body: energy, metabolism, and regulation. Annu Rev Entomol 55:207–225. 10.1146/annurev-ento-112408-085356 19725772 10.1146/annurev-ento-112408-085356 PMC3075550

[CR6] Asakura K (1978) Phosphatase activity in the larva of the euryhaline mosquito, Aëdes togoi Theobald, with special reference to sea-water adaptation. J Exp Mar Bio Ecol 31:325–337. 10.1016/0022-0981(78)90067-9 10.1016/0022-0981(78)90067-9

[CR901] Binning RR, Coats J, Kong X, Hellmich RL (2015) Susceptibility to Bt proteins is not required for Agrotis ipsilon aversion to Bt maize. Pest Manag Sci 71(4):601–606. 10.1002/ps.3901 10.1002/ps.3901 PMC4407924 25186105

[CR9] Bischof C (1995) Effects of heavy metal stress on carbohydrate and lipid concentrations in the haemolymph and total body tissue of parasitized *Lymantria**dispar* L. larvae (Lepidoptera). Comp Biochem Physiol C Pharmacol Toxicol Endocrinol 112(1):87–92. 10.1016/0742-8413(95)00079-8 10.1016/0742-8413(95)00079-8

[CR11] Blaylock MJ, Huang JW (2000) Phytoextraction of metals. In: Raskin I, Ensley BD (eds) Phytoremediation of toxic metals using plants to clean-up the environment. Wiley, New York, pp 53–70

[CR12] Bradford MM (1976) A rapid and sensitive method for the quantitation of microgram quantities of protein utilizing the principle of protein-dye binding. Anal Biochem 72:248–254. 10.1016/0003-2697(76)90527-3 942051 10.1016/0003-2697(76)90527-3

[CR13] Bream AS (2003) Laboratory evaluation of heavy metals stress on certain biochemical parameters of the aquatic insect, *Sphaerodema urinator* Duf. (Hemiptera: Belostomatidae). Commun Agric Appl Biol Sci 68:291–297 15149122

[CR14] Byung-Sik S, Choi RN, Lee CU (2001) Effects of cadmium on total lipid content and fatty acids of the greater wax moth, *Galleria mellonella*. Korean J Ecol 24:349–352

[CR15] CABI (2021) *Agrotis ipsilon* (black cutworm). CABI Compendium. 10.1079/cabicompendium.3801 10.1079/cabicompendium.3801

[CR16] Carey C, Bryant CJ (1995) Possible interrelations among environmental toxicants, amphibian development, and decline of amphibian populations. Environ Health Perspect 103:13–17. 10.1289/ehp.103-1519280 PMC1519280 7556018

[CR102] Capinera JL (2008) Black Cutworm, Agrotis ipsilon (Hufnagel)(Lepidoptera: Noctuidae). In: Capinera JL (eds). Encyclopedia of Entomology, vol 19, pp 48-49. Springer, Dordrecht. 10.1108/09504120510622904

[CR17] Chalaris M, Gkika DA, Tolkou AK, Kyzas GZ (2023) Advancements and sustainable strategies for the treatment and management of wastewaters from metallurgical industries: an overview. Environ Sci Pollut Res 30(57):119627–119653. 10.1007/s11356-023-30891-0 10.1007/s11356-023-30891-0 PMC10697902 37962753

[CR19] Chary NS, Kamala CT, Raj DSS (2008) Assessing risk of heavy metals from consuming food grown on sewage irrigated soils and food chain transfer. Ecotoxicol Environ Saf 69(3):513–524. 10.1016/j.ecoenv.2007.04.013 17555815 10.1016/j.ecoenv.2007.04.013

[CR20] Devkota B, Schmidt GH (2000) Accumulation of heavy metals in food plants and grasshoppers from the taigetos mountains. Greece Agric Ecosyst Environ 78(1):85–91. 10.1016/s0167-8809(99)00110-3 10.1016/s0167-8809(99)00110-3

[CR21] Ding P, Zhuang P, Li Z, Xia H, Lu H (2013) Accumulation and detoxification of cadmium by larvae of *Prodenia litura* (Lepidoptera: Noctuidae) feeding on Cd-enriched amaranth leaves. Chemosphere 91(1):28–34. 10.1016/j.chemosphere.2012.11.038 23276459 10.1016/j.chemosphere.2012.11.038

[CR22] Drevnick PE, Sandheinrich MB (2003) Effects of dietary methylmercury on reproductive endocrinology of fathead minnows. Environ Sci Technol 37(19):4390–4396. 10.1021/es034252m 14572090 10.1021/es034252m

[CR23] Ebau W, Rawi CS, Din Z, Al-Shami SA (2012) Toxicity of cadmium and lead on tropical midge larvae, Chironomus kiiensis Tokunaga and Chironomus javanus Kieffer (Diptera: Chironomidae). Asian Pac J Trop Biomed 2(8):631–634 23569984 10.1016/S2221-1691(12)60110-5 PMC3609360

[CR24] El-Saeady AE, Hussein AR, Hasan MY, Badr M (2015) Identification of the egyptian species of cutworm genera *Agrotis*, *Noctua* and *Scotia* (Lepidoptera–Noctuidae) based on male and female genitalia. Egypt J Agric Res 93(4):1033–1053. 10.21608/ejar.2015.156349 10.21608/ejar.2015.156349

[CR25] Emre I, Kayis T, Coskun M, Dursun O, Cogun HY (2013) Changes in antioxidative enzyme activity, glycogen, lipid, protein, and malondialdehyde content in cadmium-treated *Galleria mellonella* larvae. Ann Entomol Soc Am 106(3):371–377. 10.1603/an12137 10.1603/an12137

[CR26] Farombi EO, Adelowo OA, Ajimoko YR (2007) Biomarkers of oxidative stress and heavy metal levels as indicators of environmental pollution in African catfish (Clarias gariepinus) from nigeria ogun river. Int J Environ Res Public Health 4:158–165. 10.3390/ijerph2007040011 17617680 10.3390/ijerph2007040011 PMC3728582

[CR27] Fountain MT, Hopkin SP (2001) Continuous monitoring of *Folsomia candida* (Insecta: Collembola) in a metal exposure test. Ecotoxicol Environ Saf 48:275–286. 10.1006/eesa.2000.2007 11222037 10.1006/eesa.2000.2007

[CR28] Galal TM, Shehata HS (2015) Bioaccumulation and translocation of heavy metals by *Plantago major* L. grown in contaminated soils under the effect of traffic pollution. Ecol Indic 48:244–251. 10.1016/j.ecolind.2014.08.013 10.1016/j.ecolind.2014.08.013

[CR29] Gall JE, Boyd RS, Rajakaruna N (2015) Transfer of heavy metals through terrestrial food webs: a review. Environ Monit Assess 187:201. 10.1007/s10661-015-4436-3 25800370 10.1007/s10661-015-4436-3

[CR30] Gavrilović A (2017) Effects of Benzo[a]Pyrene dietary intake to antioxidative enzymes of *Lymantria Dispar* (Lepidoptera: Lymantriidae) larvae from unpolluted and polluted forests. Chemosphere 179:10–19. 10.1016/j.chemosphere.2017.03.083 28355530 10.1016/j.chemosphere.2017.03.083

[CR31] Haque SE, Shahriar MM, Nahar N, Haque MdS (2022) Impact of brick kiln emissions on soil quality: a case study of Ashulia brick kiln cluster. Bangladesh Environ Chall 9:100640. 10.1016/j.envc.2022.100640 10.1016/j.envc.2022.100640

[CR32] Hermes-Lima M, Zenteno-Savın T (2002) Animal response to drastic ´ changes in oxygen availability and physiological oxidative stress. Comp Biochem Physiol C Toxicol Pharmacol 133(4):537–556. 10.1016/S1532-0456(02)00080-7 12458182 10.1016/S1532-0456(02)00080-7

[CR33] Hossain MB, Masum Z, Rahman MS, Yu J, Noman MA, Jolly YN, Begum BA, Paray BA, Arai T (2022) Heavy metal accumulation and phytoremediation potentiality of some selected Mangrove species from the world’s largest mangrove forest. Biology 11(8):1144. 10.3390/biology11081144 36009771 10.3390/biology11081144 PMC9405028

[CR35] Huang D, Kong J, Seng Y (2012) Effects of the heavy metal Cu^2+^ on growth, development, and population dynamics of *Spodoptera litura* (Lepidoptera: Noctuidae). J Econ Entomol 105:288–294. 10.1603/EC11163 22420282 10.1603/EC11163

[CR36] Ilijin L, Periac-Mataruga V, Radojicic R, Lazarevic J, Nenadovic V, Vlahovic M, Mrdakovic M (2009) Effects of cadmium on protocerebral neurosecretory neurons and fitness components in *Lymantria dispar* L. Folia Biol (krakow) 58(1–2):91–99. 10.3409/fb58_1-2.91-99 10.3409/fb58_1-2.91-99 20420202

[CR38] Itoyama K, Kawahira Y, Murata M, Tojo S (1999) Fluctuations of some characteristics in the common cutworm, *Spodoptera litura* (Lepidoptera: Noctuidae) reared under different diets. Appl Entomol Zool 34:315–321. 10.1303/aez.34.315 10.1303/aez.34.315

[CR39] Jaishankar M, Tseten T, Anbalagan N, Mathew BB, Beeregowda KN (2014) Toxicity, mechanism and health effects of some heavy metals. Interdiscip Toxicol 7(2):60–72 26109881 10.2478/intox-2014-0009 PMC4427717

[CR40] Karaca A, Cetin SC, Turgay OC, Kizilkaya R (2010) Effects of heavy metals on soil enzyme activities. In: Sherameti I, Varma A (eds) Soil heavy metals, soil biology, vol 19. Springer, Heidelberg, pp 237–265. 10.1007/978-3-642-02436-8_11

[CR41] Kazimirova M, Slovak M, Manova A (1997) Host-parasitoid relationship of *Ceratitis capitata* (Diptera: Tephritidae) and *Coptera occidentalis* (Hymenoptera: Proctotrupoidea: Diapriidae) under host heavy metal stress. Eur J Entomol 94(3):409–420

[CR42] Khalid M, Hassani S, Abdollahi M (2020) Metal-induced oxidative stress: an evidence-based update of advantages and disadvantages. Curr Opin Toxicol 20–21:55–68. 10.1016/j.cotox.2020.05.006 10.1016/j.cotox.2020.05.006

[CR43] Kim B, Jeong JY, Park SH, Jung H, Kim M (2022) Effects of dietary copper sources and levels on growth performance, copper digestibility, fecal and serum mineral characteristics in growing pigs. J Anim Sci Technol 64(5):885–896 36287789 10.5187/jast.2022.e48 PMC9574621

[CR44] Krężel A, Maret W (2017) The functions of metamorphic metallothioneins in zinc and copper metabolism. Int J Mol Sci 18(6):1237 28598392 10.3390/ijms18061237 PMC5486060

[CR45] Kumar S, Zhao M, Zhang H, Rahman MA, Luo C, Rahman MM (2021) Distribution, contamination status and source of trace elements in the soil around brick kilns. Chemosphere 263:127882. 10.1016/j.chemosphere.2020.127882 32818846 10.1016/j.chemosphere.2020.127882

[CR46] Lagadic L, Caquet T, Ramade F (1994) The role of biomarkers in environmental assessment (5). Invertebr Popul Commun Ecotoxicol 3:193–208. 10.1007/BF00117084 10.1007/BF00117084 24202005

[CR47] Larsen KJ, Litsch AL, Brewer SR, Taylor DH (1994) Contrasting effects of sewage sludge and commercial fertilizer on egg to adult development of two herbivorous insect species. Ecotoxicology 3:94–109. 10.1007/bf00143408 24201933 10.1007/bf00143408

[CR48] Leung KMY, Furness RW (2001) Metallothionein induction and condition index of dog whelks *Nucella lapillus* (L.) exposed to cadmium and hydrogen peroxide. Chemosphere 44:321–325. 10.1016/S0045-6535(00)00297-6 11459135 10.1016/S0045-6535(00)00297-6

[CR49] Li L, Wang S, Shen X, Hiang M (2020) Ecological risk assessment of heavy metal pollution in the water of China’s coastal shellfish culture areas. Environ Sci Pollut Res 27:18392–18402. 10.1007/s11356-020-08173-w 10.1007/s11356-020-08173-w 32189200

[CR50] Li Y, Tan M, Wu H, Zhang A, Xu J, Meng Z, Yan S, Jiang D (2023) Transfer of Cd along the food chain: the susceptibility of *Hyphantria cunea* larvae to *Beauveria bassiana* under Cd stress. J Hazard Mater 453:131420. 10.1016/j.jhazmat.2023.131420 37084517 10.1016/j.jhazmat.2023.131420

[CR51] Lu G, Di S, Xueping Z (2011) Analysis on the current pollution situation of Cu, Pb and Zn in the cultivated black soil of songnen plain. Chin Agric Sci Bull 27(6):261–265. 10.11924/j.issn.1000-6850.2010-3430 10.11924/j.issn.1000-6850.2010-3430

[CR52] Maret W (2005) Zinc coordination environments in proteins determine zinc functions. J Trace Elem Med Biol 19(1):7–12. 10.1016/j.jtemb.2005.02.003 16240665 10.1016/j.jtemb.2005.02.003

[CR53] Mireji PO, Keating J, Hassanali A, Mbogo CM, Muturi MN, Githure JI, Beier JC (2010) Biological cost of tolerance to heavy metals in the mosquito *Anopheles gambiae*. Med Vet Entomol 24:101–107. 10.1111/j.1365-2915.2010.00863.x 20374478 10.1111/j.1365-2915.2010.00863.x PMC2921613

[CR54] Mitterbock F, Fuhrer E (1988) Effects of fluoride-polluted spruce leaves on nun moths, *Lymantria**monacha* L. (Lep Lymantriidae). J Appl Entomol 105(1):19–27

[CR55] Moura G, Vilarinho L, Machado J (2000) The action of Cd, Cu, Cr, Zn, and Pb on fluid composition of *Anodonta**cygnea* (L.): organic components. Comp Biochem Physiol Biochem Mol Biol 127(1):105–112. 10.1016/s0305-0491(00)00241-8 10.1016/s0305-0491(00)00241-8 11126745

[CR56] Newman MC (2014) Fundamentals of ecotoxicology: the science of pollution, 4th edn. CRC Press, Boca Raton. 10.1201/b17658

[CR57] Nfon E, Cousins IT, Järvinen O, Mukherjee AB, Verta M, Broman D (2009) Trophodynamics of mercury and other trace elements in a pelagic food chain from the baltic sea. Sci Total Environ 407(24):6267–6274. 10.1016/j.scitotenv.2009.08.032 19767059 10.1016/j.scitotenv.2009.08.032

[CR58] Norton KB, Kench JE (1977) Effects of cadmium on ribosomal protein synthesis in rat liver. Environ Res 13:102–110. 10.1016/0013-9351(77)90008-1 14823 10.1016/0013-9351(77)90008-1

[CR59] Notten MJ, Oosthoek AJ, Rozema J, Aerts R (2005) Heavy metal concentrations in a soil-plant-snail food chain along a terrestrial soil pollution gradient. Environ Pollut 138(1):178–190. 10.1016/j.envpol.2005.01.011 16005127 10.1016/j.envpol.2005.01.011

[CR60] Okamoto T, Taguchi M, Osaki T, Fukumoto S, Fujita T (2014) Phosphate enhances reactive oxygen species production and suppresses osteoblastic differentiation. J Bone Miner Metab 32:393–399. 10.1007/s00774-013-0516-z 24052209 10.1007/s00774-013-0516-z

[CR61] Ortel J (1991) Effects of lead and cadmium on chemical composition and total water content of the pupal parasitoid, *Pimpla turionellae*. Entomol Exp Appl 59:93–100. 10.1111/j.1570-7458.1991.tb01491.x 10.1111/j.1570-7458.1991.tb01491.x

[CR62] Phipps T, Tank SL, Wirtz J, Brewer L, Coyner A, Ortego LS, Fairbrother A (2002) Essentiality of nickel and homeostatic mechanisms for its regulation in terrestrial organisms. Environ Rev 10(4):209–261. 10.1139/a02-009 10.1139/a02-009

[CR63] Piano A, Valbonesi P, Fabbri E (2004) Expression of cytoprotective proteins, heat shock protein 70 and metallothioneins, in tissues of *Ostrea edulis* exposed to heat and heavy metals. Cell Stress Chaperones 9:134–142 15497500 10.1379/483.1 PMC1065293

[CR64] Pretorius LM (1976) Laboratory studies on the development and reproductive performance of *Heliothis armigera* (Hubn.) on various food plants. J Entomol Soc South Afr 39:337–343

[CR65] Rauschenbach IY, Bogomolova EV, Gruntenko NE, Adonyeva NV, Chentsova NA (2007) Effects of juvenile hormone and 20-hydroxyecdysone on alkaline phosphatase activity in *Drosophila* under normal and heat stress conditions. J Insect Physiol 53:587–591. 10.1016/j.jinsphys.2007.02.011 17433361 10.1016/j.jinsphys.2007.02.011

[CR66] Rodrick GE (1979) Selected enzyme activities in Mya arenaria hemolymph. Comp Biochem Physiol B 62(4):313–316. 10.1016/0305-0491(79)90095-6 95692 10.1016/0305-0491(79)90095-6

[CR67] Roe JH, Dailey RE (1966) Determination of glycogen with the anthrone reagent. Anal Biochem 15(2):245–250. 10.1016/0003-2697(66)90028-5 4289896 10.1016/0003-2697(66)90028-5

[CR68] Sancho E, Ferrando MD, Fernandez C, Andreu E (1998) Liver energy metabolism *of Anguilla Anguilla* after exposure to fenitrothion. Ecotoxicol Environ Saf 41(2):168–175. 10.1006/eesa.1998.1689 9756704 10.1006/eesa.1998.1689

[CR69] Schmidt GH, Ibrahim NM, Abdallah MD (1992) Long-term effects of heavy metals in food on developmental stages of *Aiolopus thalassinus* (Saltatoria: Acrididae). Arch Environ Contaminat Toxicol 23:375–382. 10.1007/BF00216248 10.1007/BF00216248 1456784

[CR71] Shirley MD, Sibly RM (1999) Genetic basis of a between-environment trade-off involving resistance to cadmium in *Drosophila melanogaster*. Evolution 53(3):826–836. 10.1111/j.1558-5646.1999.tb05376.x 28565631 10.1111/j.1558-5646.1999.tb05376.x

[CR72] Shu Y, Zhou J, Lu K, Li K, Zhou Q (2015) Response of the common cutworm *Spodoptera litura* to lead stress: changes in sex ratio, Pb accumulations, midgut cell ultrastructure. Chemosphere 139:441–451. 10.1016/j.chemosphere.2015.07.065 26248226 10.1016/j.chemosphere.2015.07.065

[CR73] Skuza L, Szućko-Kociuba I, Filip E, Bożek I (2022) Natural molecular mechanisms of plant hyperaccumulation and hypertolerance towards heavy metals. Int J Mol Sci 23(16):9335 36012598 10.3390/ijms23169335 PMC9409101

[CR74] Slobodian MR, Petahtegoose JD, Wallis AL, Levesque DC, Merritt TJ (2021) The effects of essential and non-essential metal toxicity in the *Drosophila melanogaster* insect model: a review. Toxics 9(10):269. 10.3390/toxics9100269 34678965 10.3390/toxics9100269 PMC8540122

[CR75] Soliman MM, Hesselberg T, Mohamed AA, Renault D (2022) Trophic transfer of heavy metals along a pollution gradient in a terrestrial agro-industrial food web. Geoderma 413:115748. 10.1016/j.geoderma.2022.115748 10.1016/j.geoderma.2022.115748

[CR76] Soliman M, Almadiy A, Al-Akeel R, Hesselberg T, Mohamed A (2024) Limited genetic variability and spatial population structure in grasshoppers between natural and metal-contaminated areas in Egypt. J Insect Sci 24(2):12. 10.1093/jisesa/ieae026 10.1093/jisesa/ieae026 PMC10949439 38501856

[CR77] Spring JH, Matthews JR, Downer RGH (1977) Fate of glucose in haemolymph of the American cockroach. Periplaneta Americana J Insect Physiol 23(4):525–529. 10.1016/0022-1910(77)90264-5 10.1016/0022-1910(77)90264-5

[CR78] Su HH, Hu MM, Harvey-Samuel T, Yang YZ (2014) Accumulation and excretion of cadmium in three successive generations of *Spodoptera exigua* (Lepidoptera: Noctuidae) and impact on the population increase. J Econ Entomol 107(1):223–229. 10.1603/EC13436 24665705 10.1603/EC13436

[CR79] Suganya M, Karthi S, Shivakumar MS (2016) Effect of cadmium and lead exposure on tissue specific antioxidant response in *Spodoptera litura*. Free Radic Antioxid 6(1):90–100. 10.5530/fra.2016.1.11 10.5530/fra.2016.1.11

[CR80] Sun H, Shu Y, Tang W, Wang Q, Zhou Q, Zhang G (2007) Nickel accumulation and its effects on the survival rate of *Spodoptera litura* Fabricius under continuous nickel stress. Chin Sci Bull 52:1957–1963. 10.1007/s11434-007-0293-y 10.1007/s11434-007-0293-y

[CR81] Sun H-X, Dang Z, Xia Q, Tang W-C, Zhang G-R (2011) The effect of dietary nickel on the immune responses of *Spodoptera litura* Fabricius larvae. J Insect Physiol 57(7):954–961. 10.1016/j.jinsphys.2011.04.008 21540035 10.1016/j.jinsphys.2011.04.008

[CR82] Takeda A (2000) Movement of zinc and its functional significance in the brain. Brain Res Rev 34:137–148. 10.1016/S0165-0173(00)00044-8 11113504 10.1016/S0165-0173(00)00044-8

[CR83] Tchounwou PB, Yedjou CG, Patlolla AK, Sutton DJ (2012) Heavy metal toxicity and the environment. Exp Suppl 101:133–164. 10.1007/978-3-7643-8340-4_6 22945569 10.1007/978-3-7643-8340-4_6 PMC4144270

[CR84] Tian J, Yang Y, Du X, Xu W, Zhu B, Huang Y, Ye Y, Zhao Y, Li Y (2023) Effects of dietary soluble β-1, 3-glucan on the growth performance, antioxidant status, and immune response of the river prawn (*Macrobrachium nipponense*). Fish Shellfish Immunol 138:108848. 10.1016/j.fsi.2023.108848 37230308 10.1016/j.fsi.2023.108848

[CR104] Tőzsér D, Magura T, Simon E, Mizser S, Papp D, Tóthmérész B (2019) Pollution intensity-dependent metal accumulation in ground beetles: a meta-analysis. Environ Sci Pollut Res 26:32092–32102. 10.1007/s11356-019-06294-5 10.1007/s11356-019-06294-5 PMC6875149 31494846

[CR85] Van Handel E (1985) Rapid determination of total lipids in mosquitoes. J Am Mosq Control Assoc 1(3):302–304 2906672

[CR86] Vickerman DB, Young JK, Trumble JT (2002) Effect of selenium-treated alfalfa on development, survival, feeding and oviposition preferences of *Spodoptera exigua* (Lepidoptera: Noctuidae). Environ Entomol 31:953–959. 10.1603/0046-225-31.6.953 10.1603/0046-225-31.6.953

[CR87] Wang Z, Liu S, Yang B, Liu Z (2011) Characterization of soluble and membrane-bound alkaline phosphatase in *Nilaparvata lugens* and their potential relation to development and insecticide resistance. Arch Insect Biochem Physiol 78:30–45. 10.1002/arch.20437 21769927 10.1002/arch.20437

[CR88] Warrington S (1987) Relationship between SO2 dose and growth of the pea aphid, *Acyrthosiphon pisum*, on peas. Environ Pollut 43(2):155–162. 10.1016/0269-7491(87)90073-x 15092808 10.1016/0269-7491(87)90073-x

[CR89] Wilps H, Gäde G (1990) Hormonal regulation of carbohydrate metabolism in the blowfly *Phormia terraenovae*. J Insect Physiol 36(6):441–449. 10.1016/0022-1910(90)90062-K 10.1016/0022-1910(90)90062-K

[CR90] Winder L, Merrington G, Green I (1999) The tri-trophic transfer of Zn from the agricultural use of sewage sludge. Sci Total Environ 229(1–2):73–81. 10.1016/S0048-9697(99)00070-4 10.1016/S0048-9697(99)00070-4

[CR91] Wu G, Ye G, Hu C, Cheng J (2006) Accumulation of cadmium and its effects on growth, development and hemolymph biochemical compositions in *Boettcherisca peregrina* larvae (Diptera: Sarcophagidae). Insect Sci 13(1):31–39. 10.1111/j.1744-7917.2006.00065.x 10.1111/j.1744-7917.2006.00065.x

[CR92] Yousef HA, Afify A, Hasan HM, Meguid AA (2010) DNA damage in hemocytes of *Schistocerca gregaria* (Orthoptera: Acrididae) exposed to contaminated food with cadmium and lead. Nat Sci 2:292–297. 10.4236/ns.2010.24037 10.4236/ns.2010.24037

[CR93] Yousef HA, Abdelfattah EA, Augustyniak M (2017) Evaluation of oxidative stress biomarkers in *Aiolopus thalassinus* (Orthoptera: Acrididae) collected from areas polluted by the fertilizer industry. Ecotoxicology 26:340–350. 10.1007/s10646-017-1767-6 28116642 10.1007/s10646-017-1767-6

[CR94] Yousef HA, Abdelfattah EA, Augustyniak M (2019) Antioxidant enzyme activity in responses to environmentally induced oxidative stress in the 5^th^ instar nymphs of *Aiolopus thalassinus* (Orthoptera: Acrididae). Environ Sci Pollut Res 26:3823–3833. 10.1007/s11356-018-3756-6 10.1007/s11356-018-3756-6 30539392

[CR95] Zhang Y, Sun G, Yang M, Wu H, Zhang J, Song S, Ma E, Guo Y (2011) Chronic accumulation of cadmium and its effects on antioxidant enzymes and malondialdehyde in *Oxya chinensis* (Orthoptera: Acridoidea). Ecotoxicol Environ Saf 74(5):1355–1362 21435721 10.1016/j.ecoenv.2011.03.002

[CR96] Zhang C, Song N, Zeng GM, Jiang M, Zhang JC, Hu XJ, Chen AW, Zhen JM (2014) Bioaccumulation of zinc, lead, copper, and cadmium from contaminated sediments by native plant species and *Acrida cinerea* in South China. Environ Monit Assess 186(3):1735–1745. 10.1007/s10661-013-3489-4 24249249 10.1007/s10661-013-3489-4

[CR97] Zhang J, Jiang D, Dong X, Meng Z, Yan S (2020) Accumulation of Cd and Pb in various body parts, organs and tissues of *Lymantria dispar asiatica* (Lepidoptera: Erebidae). J Asia Pac Entomol 23(4):963–969. 10.1016/j.aspen.2020.07.019 10.1016/j.aspen.2020.07.019

[CR98] Zhao Y, Fang X, Mu Y, Cheng Y, Ma Q, Nian H, Yang C (2014) Metal pollution (Cd, Pb, Zn, and As) in agricultural soils and soybean, Glycine max, in southern China. Bull Environ Contam Toxicol 92(4):427–432. 10.1007/s00128-014-1218-5 24519477 10.1007/s00128-014-1218-5

[CR99] Zheng D, Wang Q, Zhang Z, Zheng N, Zhang X (2008) Bioaccumulation of total and methyl mercury by arthropods. Bull Environ Contam Toxicol 81(1):95–100. 10.1007/s00128-008-9393-x 18365125 10.1007/s00128-008-9393-x

[CR100] Zhu H, Zhang J, Kim MT, Boison A, Sedykh A, Moran K (2014) Big data in chemical toxicity research: the use of high-throughput screening assays to identify potential toxicants. Chem Res Toxicol 27(10):1643–1651. 10.1021/tx500145h 25195622 10.1021/tx500145h PMC4203392

[CR101] Zvereva E, Serebrov V, Glupov V (2003) Activity and heavy metal resistance of nonspecific esterases in leaf beetle, *Chrysomela lapponica* from polluted and unpolluted habitats. Comp Biochem Physiol C Toxicol Pharmacol 135:383–391. 10.1016/S1532-0456(03)00115-7 12965183 10.1016/S1532-0456(03)00115-7

